# P-1431. Electronic Nudges to Increase Influenza Vaccination in Immunocompromised Individuals: a secondary analysis of the NUDGE-FLU-CHRONIC trial

**DOI:** 10.1093/ofid/ofaf695.1618

**Published:** 2026-01-11

**Authors:** Anne Marie Reimer Jensen, Niklas D Johansen, Lisa S Duus, Muthiah Vaduganathan, Ankeet S Bhatt, Daniel Modin, Safia Chatur, Brian L Claggett, Kira Janstrup, Joshua A Hill, Carsten S Larsen, Lykke Larsen, Lothar Wiese, Michael Dalager-Pedersen, Lars Køber, Scott D Solomon, Pradeesh Sivapalan, Jens Ulrik Jensen, Cyril Jean-Marie Martel, Tyra G Krause, Tor Biering-Sørensen

**Affiliations:** Copenhagen University Hospital - Herlev and Gentofte, Copenhagen, Hovedstaden, Denmark; Copenhagen University Hospital - Herlev and Gentofte, Copenhagen, Denmark, Copenhagen, Hovedstaden, Denmark; Copenhagen University Hospital - Herlev and Gentofte, Copenhagen, Denmark, Copenhagen, Hovedstaden, Denmark; Brigham and Women’s Hospital, Harvard Medical School, Boston, MA, USA, Boston, Massachusetts; Kaiser Permanente San Francisco Medical Center & Division of Research, San Francisco, CA, USA, San Francisco, California; Copenhagen University Hospital - Herlev and Gentofte, Copenhagen, Denmark, Copenhagen, Hovedstaden, Denmark; Brigham and Women’s Hospital, Harvard Medical School, Boston, MA, USA, Boston, Massachusetts; Brigham and Women’s Hospital, Harvard Medical School, Boston, MA, USA, Boston, Massachusetts; Copenhagen University Hospital - Herlev and Gentofte, Copenhagen, Denmark, Copenhagen, Hovedstaden, Denmark; Fred Hutchinson Cancer Center; University of Washington, Seattle, Washington; Aarhus University Hospital, Aarhus, Midtjylland, Denmark; Odense University Hospital, Odense, Syddanmark, Denmark; Roskilde Hospital, Roskilde, Sjelland, Denmark; Aalborg University Hospital, Aalborg, Nordjylland, Denmark; Copenhagen University Hospital – Rigshospitalet, Copenhagen, Hovedstaden, Denmark; Brigham and Women’s Hospital, Harvard Medical School, Boston, MA, USA, Boston, Massachusetts; Copenhagen University Hospital - Herlev and Gentofte, Copenhagen, Hovedstaden, Denmark; Copenhagen University Hospital - Herlev and Gentofte, Copenhagen, Denmark, Copenhagen, Hovedstaden, Denmark; SSI, Copenhagen, Hovedstaden, Denmark; Statens Serum Institut, Copenhagen, Denmark, Copenhagen, Hovedstaden, Denmark; Department of Cardiology, Herlev and Gentofte Hospital, Copenhagen, Hovedstaden, Denmark

## Abstract

**Background:**

Immunosuppressed individuals are at higher risk for influenza-related complications, yet vaccination rates remain low, particularly among young and middle-aged adults. This secondary analysis of the NUDGE-FLU-CHRONIC trial assessed the impact of letter-based nudges on influenza vaccine uptake by immunosuppression status.

**Table 1:**
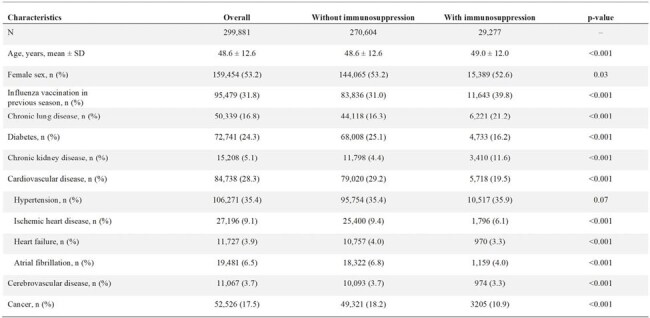
Baseline Characteristics by Immunosuppression Status

**Table 2:**
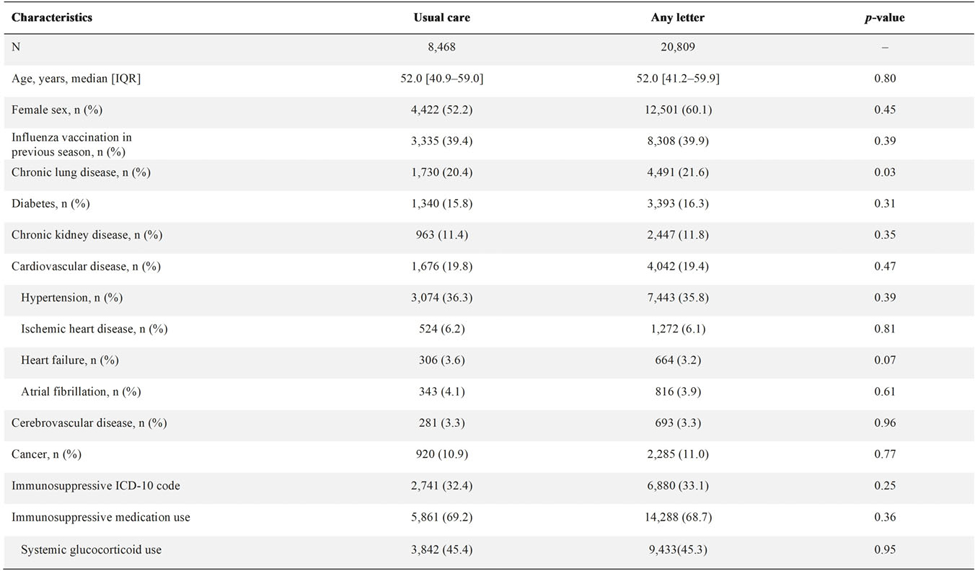
Baseline Characteristics by Randomization to Any Letter or Usual Care in Participants with Immunosuppression

**Methods:**

All Danish citizens aged 18–64 with chronic conditions eligible for free influenza vaccination during the 2023/24 season were enrolled. Participants were randomly assigned to no letter (usual care), or one of six electronic nudges delivered via the national governmental electronic letter system. All data were obtained from national registries.

Immunosuppression was defined as a diagnosis of HIV, congenital immunodeficiency, or transplantation ≤10 years before randomization, or ≥1 filled prescription for an immunosuppressant or systemic glucocorticoid ≤180 days before randomization.

The primary endpoint was influenza vaccination by January 1, 2024. Vaccination rates were compared using χ² tests and presented as absolute differences in proportions. Effect modification by immunosuppression status was assessed using binomial regression models with interaction terms.

**Figure 1:**
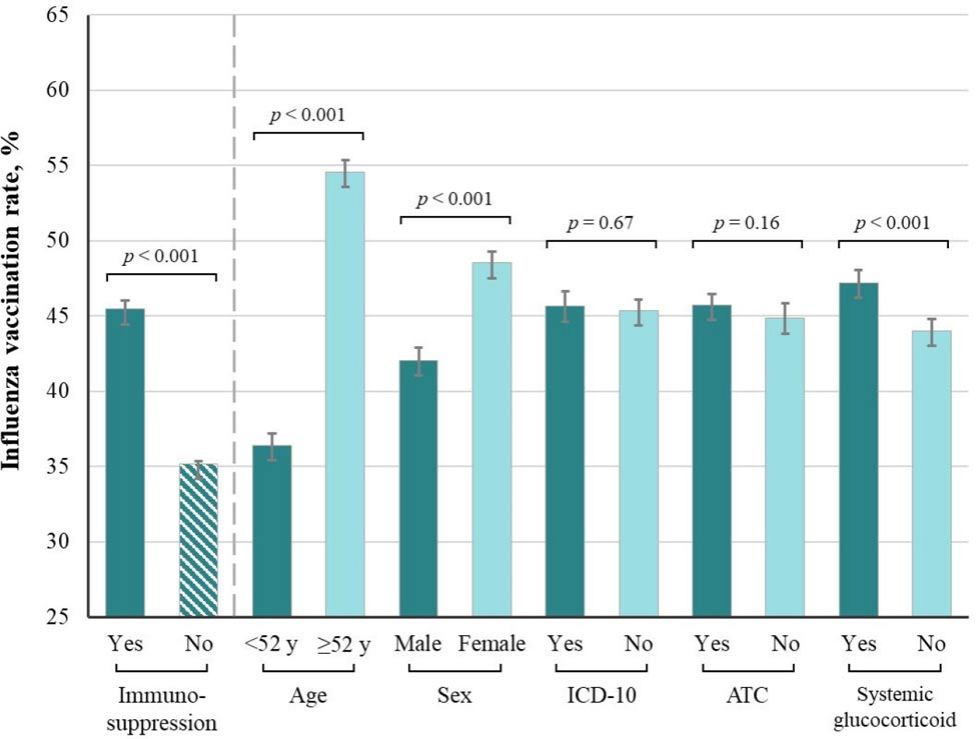
Vaccination rates by immunosuppression status Bar chart with 95% confidence intervals of influenza vaccination rates according to immunosuppression status (green vs patterned) in all NUDGE-FLU-CHRONIC participants (n=299,881), and among immunosuppression subgroups (green vs teal) among participants with immunosuppression (n=29,277). ICD-10 codes for immunsuppression included diagnosis of HIV (B20-B24, O98.7, Z21), congenital immunodeficiency (D80-D84, D89) or transplantation (Z94.0-Z94.4, Z94.8A). ATC codes for immunosuppressive medications included filled prescriptions for immunosuppressants (L04) or systemic glucocorticoids (H02AB).

**Figure 2.**
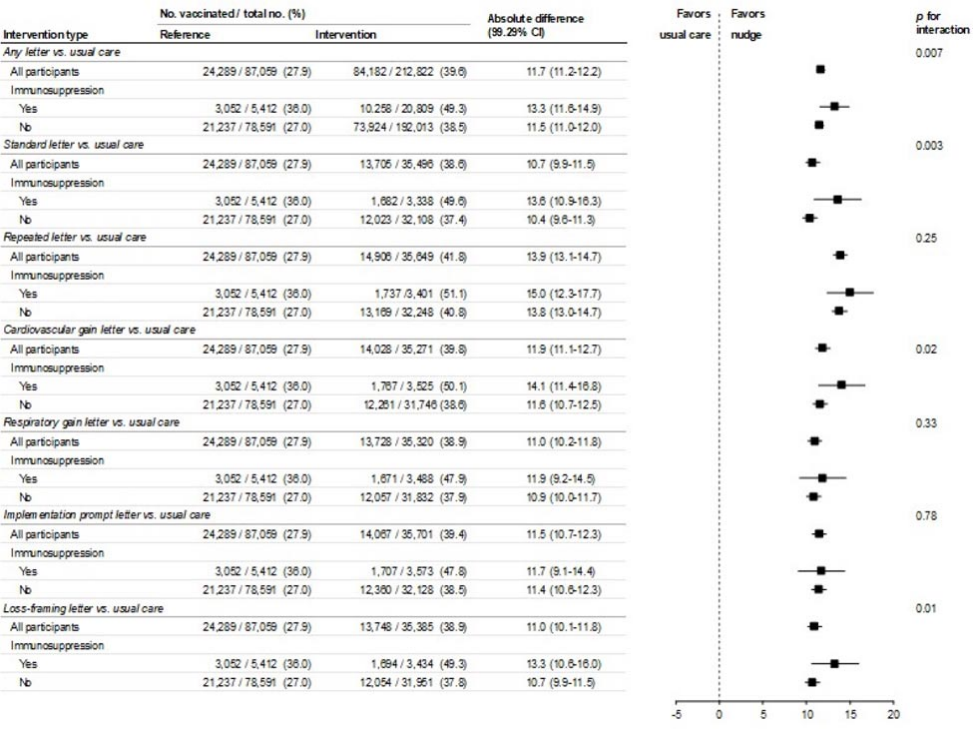
Effectiveness of Electronically Delivered Behavioral Nudges by Immunosuppression Status

**Results:**

Among 299,881 participants (median age 52 years, 53% female), 29,277 (10%) were immunosuppressed. Immunosuppressed individuals were more likely to have chronic lung or kidney disease but had lower rates of diabetes, cardiovascular disease, and cancer (Table 1). Baseline characteristics were balanced by randomization group among immunosuppressed individuals (Table 2).

Vaccination rates were higher in individuals with immunosuppression than those without (45% vs. 35%, p < 0.001; Figure 1). Any letter-based nudge improved vaccine uptake more in individuals with vs. without immunosuppression (+13.3 vs. +11.5 percentage points; *p* for interaction = 0.007), with similar patterns for the standard letter, cardiovascular-gain frame and loss-framing interventions, but no effect modification for other letter types (Figure 2).

**Conclusion:**

Electronically delivered letter-based nudges effectively increased influenza vaccination compared with usual care among individuals with immunosuppression.

**Disclosures:**

Muthiah Vaduganathan, MD, MPH, American Regent: Grant/Research Support|Amgen: Grant/Research Support|AstraZeneca: Advisor/Consultant|AstraZeneca: Grant/Research Support|Baxter Healthcare: Grant/Research Support|Bayer AG: Advisor/Consultant|Bayer AG: Grant/Research Support|BMS: Grant/Research Support|Boehringer Ingelheim: Grant/Research Support|Chiesi: Grant/Research Support|Cytokinetics: Grant/Research Support|Fresenius Medical Care: Grant/Research Support|Galmed: Advisor/Consultant|Idorsia Pharmaceuticals: Grant/Research Support|Impulse Dynamics: Advisor/Consultant|Lexicon Pharmaceuticals: Grant/Research Support|Merck: Grant/Research Support|Milestone Pharmaceuticals: Grant/Research Support|Novartis: Advisor/Consultant|Novartis: Grant/Research Support|Novo Nordisk: Advisor/Consultant|Novo Nordisk: Grant/Research Support|Occlutech: Advisor/Consultant|Pharmacosmos: Grant/Research Support|Relypsa: Grant/Research Support|Roche Diagnostics: Grant/Research Support|Sanofi: Grant/Research Support|Tricog Health: Grant/Research Support Ankeet S. Bhatt, MD, MBA, ScM, Merck: Honoraria|Novo Nordisk: Honoraria|Sanofi: Honoraria Brian L. Claggett, PhD, Alnylam: Advisor/Consultant|Cardior: Advisor/Consultant|Cardurion: Advisor/Consultant|CVRx: Advisor/Consultant|Cytokinetics: Advisor/Consultant|Eli Lilly: Advisor/Consultant|Intellia: Advisor/Consultant|Rocket: Advisor/Consultant Joshua A. Hill, MD, Allovir: Advisor/Consultant|Allovir: Grant/Research Support|CSL Behring: Advisor/Consultant|Gilead Sciences: Advisor/Consultant|Gilead Sciences: Grant/Research Support|Karius: Advisor/Consultant|Merck: Grant/Research Support|Moderna: Advisor/Consultant|Takeda: Advisor/Consultant|Takeda: Grant/Research Support Carsten S. Larsen, MD, DMSc, Danske Lægers Vaccinations Service: Employed as chief physician|GSK: Advisor/Consultant|MSD: Advisor/Consultant|Pfizer: Advisor/Consultant|Takeda: Advisor/Consultant|Valneva: Advisor/Consultant Lars Køber, MD, DMSc, AstraZeneca: Honoraria|Bayer: Honoraria|Boehringer Ingelheim: Honoraria|Novartis: Honoraria|Novo Nordisk: Honoraria Scott D. Solomon, MD, Abbott: Advisor/Consultant|Action: Advisor/Consultant|Akros: Advisor/Consultant|Alexion: Advisor/Consultant|Alexion: Grant/Research Support|Alnylam: Advisor/Consultant|Alnylam: Grant/Research Support|Amgen: Advisor/Consultant|Applied Therapeutics: Grant/Research Support|Arena: Advisor/Consultant|AstraZeneca: Advisor/Consultant|AstraZeneca: Grant/Research Support|Bayer: Advisor/Consultant|Bayer: Grant/Research Support|Bellerophon: Grant/Research Support|BMS: Advisor/Consultant|BMS: Grant/Research Support|Boston Scientific: Grant/Research Support|Cardior: Advisor/Consultant|Cardurion: Advisor/Consultant|Corvia: Advisor/Consultant|Cytokinetics: Advisor/Consultant|Cytokinetics: Grant/Research Support|Edgewise: Grant/Research Support|Eidos/BridgeBio: Grant/Research Support|Gossamer: Grant/Research Support|GSK: Advisor/Consultant|GSK: Grant/Research Support|Ionis: Grant/Research Support|Lilly: Advisor/Consultant|Lilly: Grant/Research Support|Moderna: Advisor/Consultant|Novartis: Advisor/Consultant|Novartis: Grant/Research Support|NovoNordisk: Grant/Research Support|Quantum Genomics: Advisor/Consultant|Respicardia: Grant/Research Support|Sanofi Pasteur: Advisor/Consultant|Sanofi Pasteur: Grant/Research Support|Tenaya: Advisor/Consultant|Tenaya: Grant/Research Support|Theracos: Advisor/Consultant|Theracos: Grant/Research Support|US2.AI: Grant/Research Support Tor Biering-Sørensen, MD, PhD, Amgen: Advisor/Consultant|AstraZeneca: Grant/Research Support|AstraZeneca: Honoraria|Bayer: Grant/Research Support|Bayer: Honoraria|Boston Scientific: Grant/Research Support|CSL Seqirus: Advisor/Consultant|GE Healthcare: Grant/Research Support|GE Healthcare: Honoraria|GSK: Advisor/Consultant|GSK: Grant/Research Support|GSK: Honoraria|IQVIA: Advisor/Consultant|Novartis: Grant/Research Support|Novartis: Honoraria|Novo Nordisk: Advisor/Consultant|Novo Nordisk: Grant/Research Support|Parexel: Advisor/Consultant|Pfizer: Grant/Research Support|Sanofi Pasteur: Advisor/Consultant|Sanofi Pasteur: Grant/Research Support|Sanofi Pasteur: Honoraria

